# Window-of-Opportunity Study of Valproic Acid in Breast Cancer Testing a Gene Expression Biomarker

**DOI:** 10.1200/PO.16.00011

**Published:** 2017-04-07

**Authors:** Adam L. Cohen, Leigh Neumayer, Ken Boucher, Rachel E. Factor, Gajendra Shrestha, Mark Wade, John G. Lamb, Kylee Arbogast, Stephen R. Piccolo, Joanna Riegert, Matthias Schabel, Andrea H. Bild, Theresa L. Werner

**Affiliations:** **Adam L. Cohen**, **Leigh Neumayer**, **Ken Boucher**, **Rachel E. Factor**, **Gajendra Shrestha**, **John G. Lamb**, **Stephen R. Piccolo**, **Andrea H. Bild**, and **Theresa L. Werner**, University of Utah; **Adam L. Cohen**, **Mark Wade**, **Kylee Arbogast**, **Joanna Riegert**, and **Theresa L. Werner**, Huntsman Cancer Institute, Salt Lake City; **Stephen R. Piccolo**, Brigham Young University, Provo, UT; **Leigh Neumayer**, University of Arizona, Tucson, AZ; and **Matthias Schabel**, Advanced Imaging Research Center, Portland, OR

## Abstract

**Purpose:**

The anticancer activity of valproic acid (VPA) is attributed to the inhibition of histone deacetylase. We previously published the genomically derived sensitivity signature for VPA (GDSS-VPA), a gene expression biomarker that predicts breast cancer sensitivity to VPA in vitro and in vivo. We conducted a window-of-opportunity study that examined the tolerability of VPA and the ability of the GDSS-VPA to predict biologic changes in breast tumors after treatment with VPA.

**Patients and Methods:**

Eligible women had untreated breast cancer with breast tumors larger than 1.5 cm. After a biopsy, women were given VPA for 7 to 12 days, increasing from 30 mg/kg/d orally divided into two doses per day to a maximum of 50 mg/kg/d. After VPA treatment, serum VPA level was measured and then breast surgery or biopsy was performed. Tumor proliferation was assessed by using Ki-67 immunohistochemistry. Histone acetylation of peripheral blood mononuclear cells was assessed by Western blot. Dynamic contrast-enhanced magnetic resonance imaging scans were performed before and after VPA treatment.

**Results:**

Thirty women were evaluable. The median age was 54 years (range, 31-73 years). Fifty-two percent of women tolerated VPA at 50 mg/kg/d, but 10% missed more than two doses as a result of adverse events. Grade 3 adverse events included vomiting and diarrhea (one patient) and fatigue (one patient). The end serum VPA level correlated with a change in histone acetylation of peripheral blood mononuclear cells (ρ = 0.451; *P* = .024). Fifty percent of women (three of six) with triple-negative breast cancer had a Ki-67 reduction of at least 10% compared with 17% of other women. Women whose tumors had higher GDSS-VPA were more likely to have a Ki-67 decrease of at least 10% (area under the curve, 0.66).

**Conclusion:**

VPA was well tolerated and there was a significant correlation between serum VPA levels and histone acetylation. VPA treatment caused a decrease in proliferation of breast tumors. The genomic biomarker correlated with decreased proliferation. Inhibition of histone deacetylase is a valid strategy for drug development in triple-negative breast cancer using gene expression biomarkers.

## INTRODUCTION

The goal of breast cancer management is to provide personalized therapy. Currently, patients receive a series of sequential chemotherapies, antiestrogen therapies, and/or receptor-targeting drugs.^1^ Treatment with nonspecific chemotherapy has substantial toxicities. Treatment individualization has been accomplished to a limited extent with the use of hormone receptor testing to determine a patient’s eligibility for antiestrogen therapy and human epidermal growth factor receptor 2 (HER2) testing to determine eligibility for anti-HER2 therapies. However, with the ability to analyze tumors on the basis of their genomic profiles, further individualization may be possible for additional cancer therapies.

Histone deacetylase (HDAC) inhibitors have shown promise in breast cancer in vitro, although this promise has not yet translated to clinical benefit. HDAC inhibitors have multiple cellular effects, including increasing the expression of tumor suppression genes,^2^ increasing the expression of cell cycle regulators,^3^ increasing the expression of mediators of apoptosis,^4-7^ decreasing proteasome-mediated degradation of tumor suppressor genes,^8^ decreasing oncoprotein levels via loss of hsp90-mediated protection,^9-11^ decreasing mitotic stability,^12^ and decreasing angiogenesis.^13,14^ HDAC inhibitors potentiate the apoptotic effect of anthracyclines on breast cancer cell lines.^15^

Valproic acid (VPA) is an antiepileptic discovered in 1882, which has been used clinically since 1962. VPA inhibits class I and class IIa HDACs.^16,17^ In vitro and in vivo studies show that VPA at clinically relevant concentrations inhibits the proliferation of breast cancer cell lines and xenografts.^18-21^ VPA has been used in breast cancer in combination with chemotherapy in three small trials^22-24^ in which response rates ranged from 33% to 70%, but the relative contribution of VPA and other drugs could not be determined. By incorporating prior knowledge about the underlying signaling pathways that drive cancer progression, we can help define the patient subgroups that may benefit from VPA. We previously published a gene expression signature that predicted the sensitivity of breast cancer to VPA in vitro and in vivo.^21^ We refer to this signature as the genomically derived sensitivity signature for VPA (GDSS-VPA).

Before committing to phase I and II studies, it is important to establish the biologic effect of a drug and get preliminary data on potential biomarkers. In window-of-opportunity studies, women with newly diagnosed breast cancer receive a drug between the diagnostic breast biopsy and planned surgical resection or the start of neoadjuvant therapy. Decrease in Ki-67 during window-of-opportunity studies is an indication of anticancer activity of the drug being studied.^25-28^ The Valproic Acid Signature Trial (VAST) was a window-of-opportunity study designed to assess the tolerability of VPA, to validate the ability of the GDSS-VPA to predict decreases in Ki-67 and tumor changes on dynamic contrast-enhanced magnetic resonance imaging (DCE-MRI) scanning, and to correlate VPA biologic activity with blood VPA levels and histone acetylation changes in blood.

## PATIENTS AND METHODS

### Study Design

VAST was a prospective, open-label, single-center, window-of-opportunity study sponsored by the Huntsman Cancer Institute and approved by the University of Utah Institutional Review Board. The primary objectives were to determine safety and tolerability of VPA, whether VPA levels correlate with histone acetylation in peripheral blood mononuclear cells (PBMCs) during treatment, and whether either VPA levels or histone acetylation in PBMCs predicts histone acetylation in tumor samples after treatment. Key secondary objectives were to assess the sensitivity and specificity of the GDSS-VPA to predict histologically measured antitumor activity of VPA in breast cancer, to assess the sensitivity and specificity of the GDSS-VPA to predict antitumor activity of VPA in breast cancer as measured by DCE-MRI scanning, and to determine whether women have dose-limiting toxicities during or after treatment with VPA over 7 to 12 days.

### Eligibility

Women with any stage of breast cancer who had received no prior treatment were potentially eligible for the trial. Adult women were eligible if they had Eastern Cooperative Oncology Group performance status of 0 to 2, a biopsy-proven invasive adenocarcinoma of the breast 1.5 cm or larger by clinical examination or imaging, were able to provide consent, and either were scheduled for breast surgery or were willing to have an end-of-study biopsy. Exclusion criteria included pregnancy; need for immediate chemotherapy; hypersensitivity to VPA or its components; peanut allergy (because some VPA formulations contain peanut oil); inadequate bone marrow, kidney, or liver function (greater than grade 1 by Common Terminology Criteria for Adverse Events v3); were immunocompromised as a result of medications or HIV; used other antiepileptics or medications that interact with VPA; had inborn errors of metabolism; had a history of pancreatitis; were on a ketogenic diet; were unable to have an MRI scan; or had a tumor that was unlikely to yield adequate tissue for genomic studies.

### Conduct of the Study

Women were enrolled between the biopsy that diagnosed their breast cancer and the start of therapy for the breast cancer, and all provided informed consent. If RNA was not available from the diagnostic biopsy, a biopsy was performed at the start of the study. One core was placed in RNAlater (ThermoFisher Scientific, Waltham, MA) for RNA isolation and one formalin-fixed paraffin-embedded core was assessed by one pathologist to confirm the presence of tumor and to assess Ki-67 (clone MIB-1; DAKO, Copenhagen, Denmark), caspase 3 (clone JHM62; Leica mMicrosystems, Wetzlar, Germany), and p21 (Clone SX118; BD Pharmingen, San Jose, CA) immunohistochemistry. Estrogen receptor (ER), progesterone receptor (PR), and HER2 were assessed by using standard diagnostic methods.^29,30^ The percent of invasive cancer cells that expressed Ki-67 in samples before and after treatment with VPA was determined manually by a certified expert pathologist (R.E.F.), who used an eyeball estimate. By using a subset of 24 slides, we compared Ki-67 by eyeballing, manual counting, and digital imaging, which had high correlation (Cronbach’s α > .9; unpublished data). A DCE-MRI scan was performed at baseline and after VPA treatment.

VPA was started at 30 mg/kg/d orally divided into two doses per day. The start of VPA treatment was designated day 1. If no intolerable grade 2 or higher adverse effects (AEs) were present, the dose was increased every 3 days by 10 mg/kg/d to a maximum of 50 mg/kg/d. The highest tolerated dose was continued until the day of surgery or the end-of-study biopsy, which was not before 14 doses (7 days) of VPA had been given or after 24 doses (12 days) of VPA had been given.

Once the patient had received the highest tolerated dose of VPA treatment for at least four doses, a DCE-MRI scan was performed followed by either surgical excision of the tumor per standard of care or a repeat core biopsy. The last dose of VPA was given on the morning of surgery or post-treatment biopsy. A core from the post-treatment biopsy or surgery was placed in RNAlater for RNA isolation, and the RNA was run on HGU133A microarrays. All microarrays were run in one batch. The blood VPA level was assessed, and PBMCs were isolated that day as well. PBMCs were frozen, and histone acetylation was assessed by using Western blot (Acetyl-H3 antibody #9649S and beta-actin antibody #3700S; Cell Signaling Technology, Danvers, MA).

The GDSS-VPA was calculated by using RNA from the pretreatment and post-treatment specimens as previously described.^21^ Parameters for the gene expression signature were locked before the trial. CEL files with the gene expression data from women on the VAST trial were anonymized by an independent statistician not otherwise involved in the trial. The CEL files have been submitted to Gene Expression Omnibus as GSE83530.

AEs were determined by using Common Terminology Criteria for Adverse Events v3. Cognitive effects were assessed by using the short portable mental status questionnaire (SPMSQ).^31^

### Statistical Analysis

A sample size of 33 patients was planned to give a power of 80% to detect a correlation of 0.5 between the VPA level on the day of surgery and change in tumor or peripheral blood histone acetylation from day 0 to the day of surgery at a two-sided α = .025. For the key end point of accuracy of the GDSS-VPA, the primary measure of antitumor activity was a decrease in Ki-67 by 20%. Accuracy of predictions was determined by using area under the receiver operating characteristic (ROC) curves calculated by using the pROC R package.^32^ CIs for ROC curves were calculated by using the method of DeLong.^33^ Thirty-three patients would result in a precision for estimating the specificity of the GDSS-VPA of ± 14% if the specificity were 0.8 and ± 17% if the specificity were 0.6. For correlating GDSS-VPA and change in Ki-67, Spearman’s ρ was used. Calculations were performed with R v3.2.1 (www.r-project.org), and figures were prepared by using GraphPad Prism 6.

## RESULTS

### Patient Characteristics

**Fig 1.**
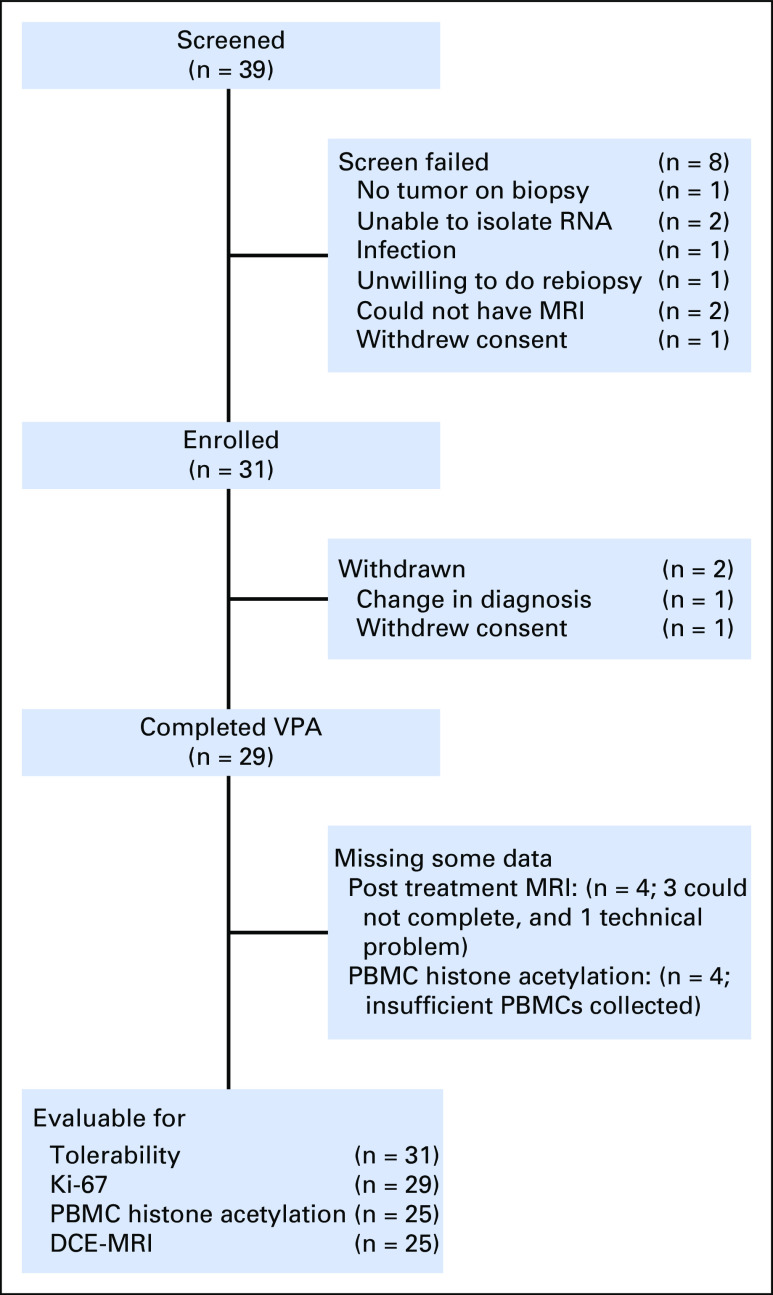
Flow of participants through the Valproic Acid Signature Trial. DCE-MRI, dynamic contrast-enhanced magnetic resonance imaging; PBMC, peripheral blood mononuclear cell; VPA, valproic acid.

Between June 2010 and August 2014, 39 patients were screened, 31 were enrolled, and 29 were treated with VPA (Fig 1). The study was stopped early because of slow accrual. The characteristics of the patients are shown in Table 1. Patient characteristics match those of the general population of breast cancer patients, with most patients having tumors that were ER-positive (64%). Nine patients (31%) had lumpectomy or mastectomy immediately after completing the study; the remaining patients received neoadjuvant chemotherapy. All patients completed the study.

**Table 1.**
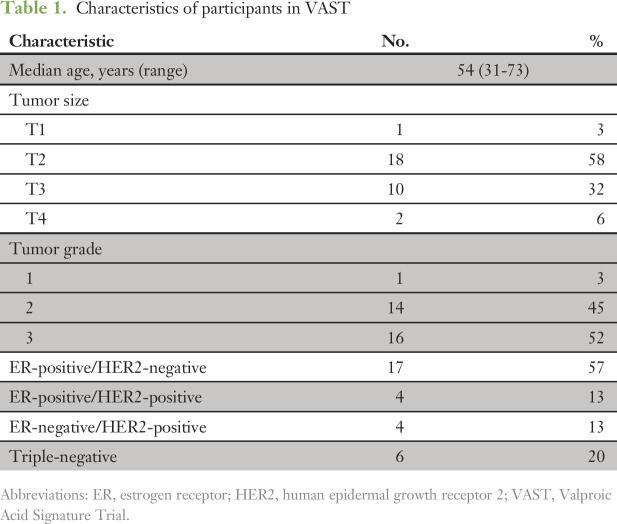
Characteristics of participants in VAST

### Safety and Tolerability

Of the 31 women who started VPA treatment, 16 (51.6%) completed the 50 mg/kg/d dose. The maximum dose tolerated was 40 mg/kg/d for 7 women (22.6%) and 30 mg/kg/d for 5 women (16.1%). Three women (9.6%) stopped VPA completely because of AEs.

The AE profile is consistent with the known effects of VPA (Table 2). The most common AEs were fatigue (80.6%), nausea (58.1%), vomiting (48.4%), dizziness (48.4%), memory impairment (32.3%), heartburn (29.0%), dysphasia (25.8%), and weakness (22.6%). The GI symptoms were manageable with antiemetics and antacids. Despite subjective feelings of cognitive impairment, all women had normal scores (two or fewer errors) on the SPMSQ throughout the study. One patient had a surgical delay for thrombocytopenia. All AEs resolved quickly upon stopping VPA.

**Table 2.**
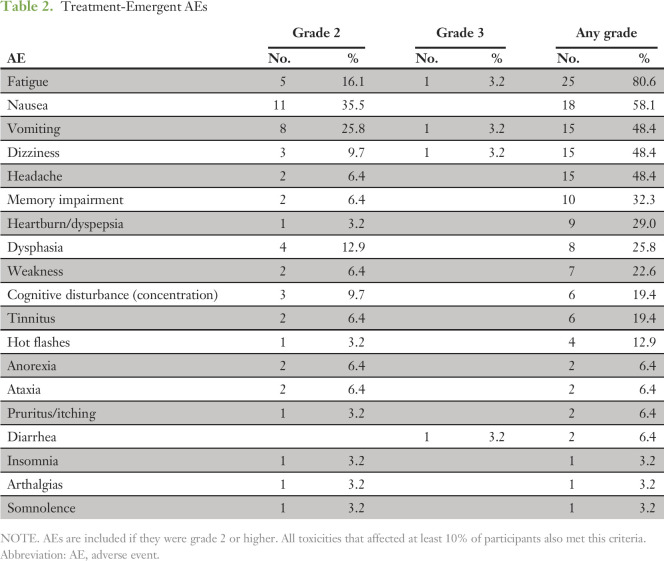
Treatment-Emergent AEs

### Pharmacodynamics

**Fig 2.**
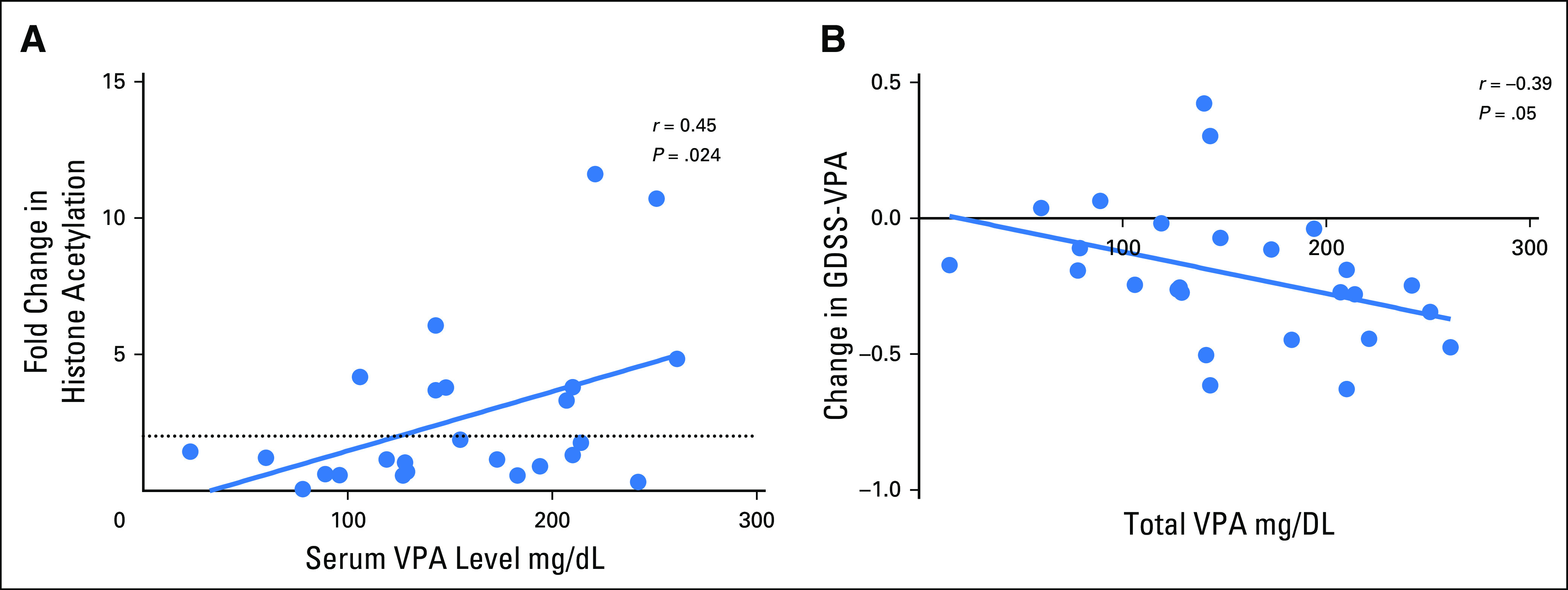
(A) Scatter plot comparing serum valproic acid (VPA) level on the last day of treatment and the change in peripheral blood mononuclear cell histone 3 acetylation. The change in histone 3 acetylation is calculated as the ratio of the normalized expression of acetylated histone 3 by Western blot on the final day of treatment to that before treatment. (B) Scatter plot comparing the total serum VPA level on the last day of treatment and the change in genomically derived sensitivity signature for VPA (GDSS-VPA).

Most women (68%) had increased PBMC histone 3 (H3) acetylation after treatment with VPA (Fig 2A). However, only 36% had more than a doubling of H3 acetylation. Similarly, 80% of women had increased levels of histone 4 acetylation, with 32% having more than a doubling. There was a statistically significant correlation between serum VPA levels on the last day of treatment and the fold change in H3 acetylation (*r* = 0.45; *P* = .024). There seemed to be a threshold effect at 100 µg/mL of VPA, because no women with VPA levels below this level had a doubling of PBMC H3 acetylation. However, change in histone acetylation was inconsistent; three of eight women with serum VPA levels above 200 µg/mL had less than a doubling of H3 acetylation. H3 acetylation in tumors could not be assessed because of technical issues.

We assessed the change in gene expression from before to after VPA treatment in breast tumors by using the GDSS-VPA, which was designed by comparing gene expression patterns in breast cancer cells before and after in vitro treatment with VPA.^21^ The gene expression pattern in the post-treatment samples was significantly more like that seen in cells treated in vitro with VPA (mean difference, –0.2063 on a scale of 0 to 1, with 0 being the most like untreated cells and 1 being the most like VPA-treated cells; *P* < .001 by paired *t* test). The change in GDSS-VPA in the post-treatment samples compared with the pretreatment samples correlated with the total VPA level (*r* = –0.39; *P* = .05; Fig 2B).

We previously published that inhibition of HDAC downregulates the MYC pathway in vitro.^3^ In the tumor samples before and after VPA treatment in the VAST trial, we examined the gene expression patterns using gene set omic analysis (GSOA) and generally applicable gene set enrichment (GAGE).^34,35^ By using either the C6 data sets (*P* = .022 by GSOA; *P* = 4.47 × 10^−7^ by GAGE) or the Hallmark pathway gene sets (*P* = .001 by GSOA; *P* = 4.42 × 10^−61^by GAGE) from MSigDB,^36^ genes regulated by MYC were downregulated in the post-treatment samples compared with the pretreatment samples.

### Effects on Tumors

**Fig 3.**
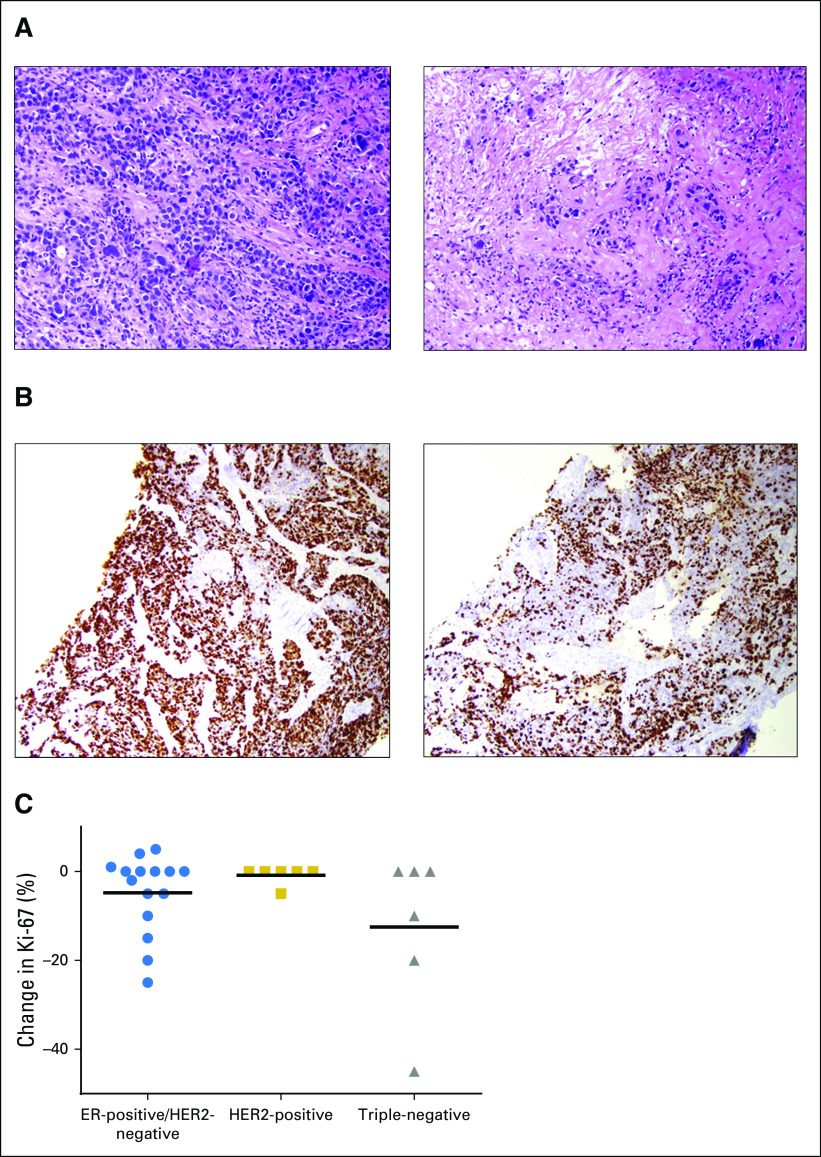
(A) Hematoxylin and eosin stain from biopsies before (left) and after (right) treatment with valproic acid (VPA) showing fibrosis typical of a post-treatment effect. (B) Ki-67 immunohistochemistry from biopsies before (left) and after (right) VPA treatment showing a decrease from approximately 70% to approximately 30%. (C) Scatter plot of the absolute change in Ki-67 from the post-treatment tumor compared with the pretreatment tumor by tumor subtype. The *y*-axis is linear. Horizontal lines show the mean change in Ki-67 for each subgroup.

Four women (14%) had a decrease in Ki-67 of at least 20%, and seven (24%) had a decrease in Ki-67 of at least 10%. Examples of histologic treatment effect and decrease in proliferation by Ki-67 immunohistochemistry are shown in Figure 3A-B. The change in Ki-67 was not related to the final VPA level. However, pretreatment tumor characteristics seemed to influence response to VPA treatment (Fig 3C). No woman with HER2-positive breast cancer had a decrease in Ki-67 of 10% or greater. Thirty-one percent of the women with ER-positive/HER2-negative breast cancer had a decrease in Ki-67 of at least 10%, and 50% of women with triple-negative breast cancer (TNBC) had a decrease in Ki-67 of 10% or greater with VPA treatment. Baseline Ki-67 also predicted change in Ki-67 (*r* = –0.37; *P* = .04).

In post hoc analyses, we compared the four women whose tumors had decrease in Ki-67 of 20% or greater with the rest of the women. There was no detectable difference in age, grade, tumor size, baseline Ki-67, final VPA level, or tumor subtype between the four responding women and the rest of the group, with the exception of the lack of HER2-positve tumors among the responders. In particular, of the four women with tumors whose Ki-67 decreased by 20% or more, two tumors were ER-positive/PR-positive and two were triple-negative, two were grade 3 and two were grade 2.

**Fig 4.**
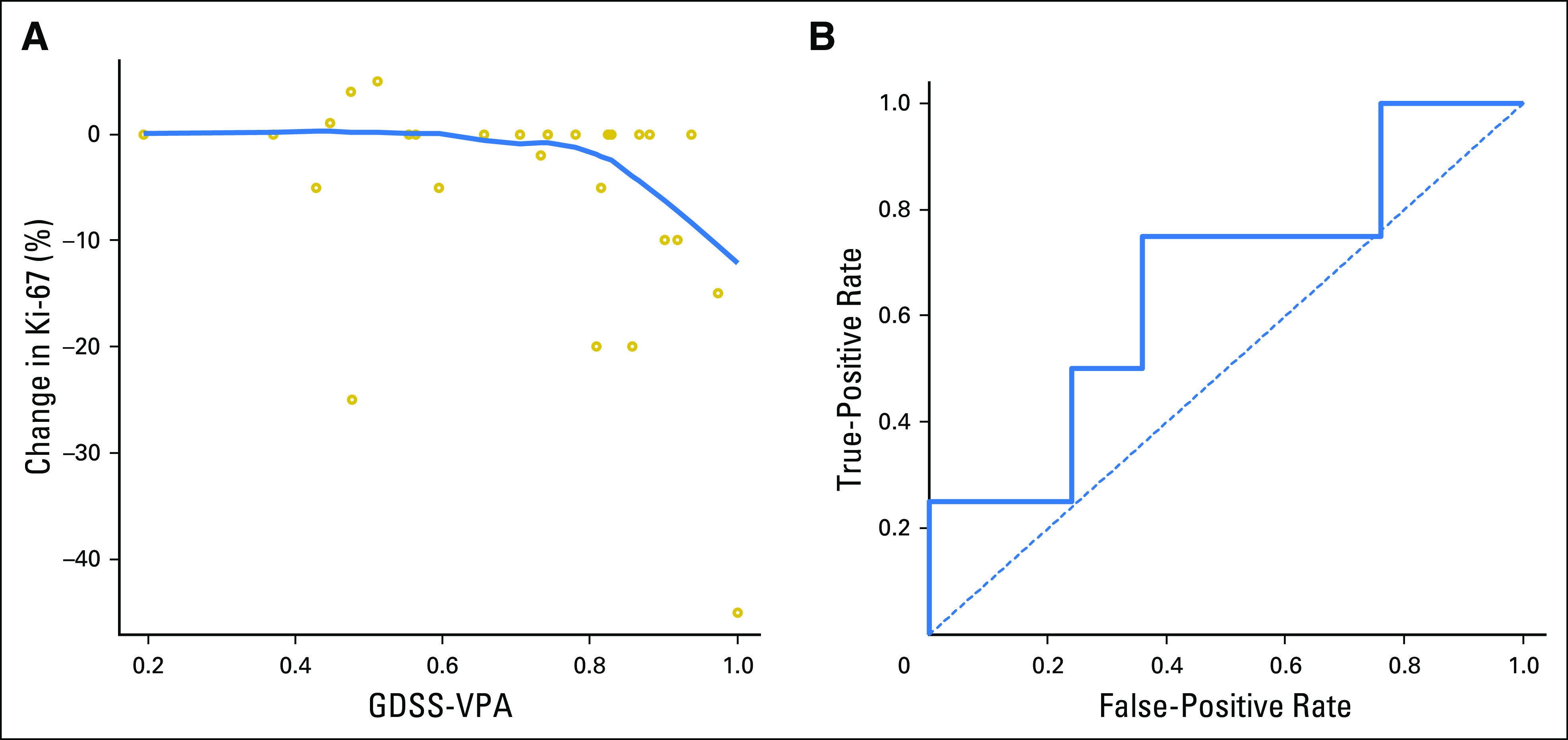
(A) Scatter plot comparing genomically derived sensitivity signature for valproic acid (GDSS-VPA) prediction to the change in Ki-67 from the pretreatment tumor to the post-treatment tumor. The prediction ranges from 0 (predicted most resistant) to 1 (predicted most sensitive). The curved line gives a LOWESS regression curve. (B) Receiver operating characteristic curve for the GDSS-VPA predictor for predicting a decrease in Ki-67 of 20%.

In addition to using GDSS-VPA as an indicator of exposure to VPA, we tested its ability as a biomarker on the pretreatment tumor RNA to predict sensitivity to VPA. There was a statistically significant relationship between the gene expression predictor GDSS-VPA and change in Ki-67 (ρ = –0.43; *P* = .021), although the relationship seemed to be nonlinear (Fig 4A). Analysis of ROC curves showed an area under the curve of 0.66 (95% CI, 0.38 to 0.95) for predicting a Ki-67 reduction of 20% with the GDSS-VPA (Fig 4B). With the optimal cutoff chosen post hoc on the basis of the ROC curves, the GDSS-VPA had a sensitivity of 75% and a specificity of 64% for predicting a decrease in Ki-67 of at least 20%. In a post-hoc analysis excluding women with HER2-positive tumors, because of the lack of response in this subgroup, the area under the curve increased to 0.74 (95% CI, 0.44 to 1.00).

As expected, given the short duration of treatment, there were no changes in tumor size between the pre- and post-treatment MRI scans. No tumors showed a change in ER expression in response to VPA treatment. One tumor, which had a decrease from 60% to 40% in Ki-67, was grade 3 on the pretreatment biopsy and grade 2 on the post-treatment lumpectomy, and one tumor was grade 2 on the pretreatment biopsy and grade 3 on the post-treatment lumpectomy. These changes are consistent with the known rate of discrepancy between biopsies and subsequent excisions. There were no significant changes in any perfusion imaging parameters on DCE-MRI with VPA treatment. Caspase 3 and p21 immunohistochemistry were not informative (data not shown).

## DISCUSSION

A window-of-opportunity trial was feasible in women with breast cancer in the United States. Window-of-opportunity studies have been proposed as an efficient use of the time between diagnosis and definitive therapy for testing the biologic effects of novel therapies. Window-of-opportunity studies in breast cancer have been completed in the United Kingdom, Sweden, and Canada.^25,26,37-41^ In the United States, window-of-opportunity studies have been conducted but have been more challenging.^42-46^ Despite the lack of evidence of harm from short delays in definitive therapy, women in the United States are often reluctant to wait more than 2 weeks between seeing a surgeon and having surgery.^41^

The VAST trial enrolled 94% of the goal population over 4 years. Although significant hurdles remain, this study demonstrates that window-of-opportunity studies can be completed for breast cancer in the United States. We found that limiting the delay to 1 week helped with accrual.

The study’s key objectives were met. VPA had no grade 4 toxicities at doses that achieved histone acetylation changes in peripheral blood, and serum VPA levels correlated with histone acetylation in PBMCs. VAST demonstrates that HDAC inhibitors have biologic activity in a subset of breast cancers. Without treatment, the absolute change in Ki-67 between biopsy and surgery averages 2% to 4%.^47^ By contrast, large changes in Ki-67 were seen in some women in our trial, particularly women with TNBC. Interestingly, no activity was seen in HER2-positive cancers. This contrasts with in vitro and in silico data from our group and from others.^21,48^ The reason for this lack of sensitivity may be related to the differences in expression of the various HDACs among breast cancer subtypes.^49,50^

Our results complement another recent window-of-opportunity study of HDAC inhibitors in breast cancer. Stearns et al^44^ treated women with 3 days of vorinostat before surgery and found a decrease in Ki-67 gene expression. The authors saw no differences in apoptosis, similar to our data. In their trial, no significant change in Ki-67 by immunohistochemistry was seen, possibly because of the shorter treatment time. They did not examine clinical, histologic, or genomic predictors of response to HDAC inhibition. Other ongoing studies are combining HDAC inhibitors with chemotherapy (NCT02632071, NCT02393794), endocrine therapy (NCT02820961, NCT02115282, NCT02395627), or immunotherapy (NCT02708680, NCT02453620, NCT02395627). None of these trials use a biomarker selected population.

The GDSS-VPA was a prespecified gene expression signature performed in a blinded fashion. Importantly, tumors that were predicted to be resistant to VPA were unlikely to have a decrease in Ki-67 in response to a short period of treatment with VPA. Alternatively, most of the tumors that were predicted to be sensitive to VPA did show decreases in Ki-67. Our results offer some support to the idea that a sensitivity biomarker based on in vitro gene expression changes can predict biologic behavior in patients. Like any biomarker, GDSS-VPA was not perfect. Discrepant response to drug and biomarker prediction could be the result of tumor heterogeneity, subtherapeutic VPA levels, deficiencies in the signature, or insufficient time of treatment; our sample size was too small for a multivariable analysis.

VPA faces significant challenges as an anti–breast cancer agent. Although by traditional measures it is well tolerated with no grade 4 toxicities, many of the grade 2 toxicities, including dizziness, sleepiness, and cognitive slowing, are intolerable over long periods of time and are difficult to ameliorate with standard supportive care. A substantial minority of women were unable to tolerate a therapeutic level of VPA. Finally, the nonlinear relationship between VPA serum levels and PBMC histone acetylation changes suggests pharmacogenetic effects that may make VPA ineffective in some people. Therefore, other HDAC inhibitors may be more appropriate for future studies.

Weaknesses of our study include the small sample size and the inability to assess histone acetylation changes in tumors. The sample size limited the power to assess the independence of variables that affected sensitivity to VPA, including tumor subtype and GDSS-VPA. Conclusions about the relationship between tumor subtype and sensitivity to VPA are hypothesis generating only. Finally, the short time period of treatment may not have been long enough to see the full effect of treatment.

We were able to complete a window-of-opportunity trial of VPA in breast cancer and to test a gene expression biomarker of sensitivity to VPA. The VAST trial showed both a biologic response of breast tumors, particularly TNBCs, to HDAC inhibition and a significant correlation between our drug response biomarker and decreased proliferation after treatment with VPA. Future studies of HDAC inhibitors in select populations testing gene expression biomarkers are warranted.
